# Reprogramming *Listeria monocytogenes* flavin metabolism to improve its therapeutic safety profile and broaden innate T-cell activation

**DOI:** 10.1128/mbio.03652-25

**Published:** 2025-12-31

**Authors:** Victoria Chevée, Mariya Lobanovska, Rafael Rivera-Lugo, Leslie Güereca, Ying Feng, Andrea Anaya-Sanchez, Jesse Garcia Castillo, Austin M. Huckins, Edward E. Lemmens, Chris S. Rae, Jonathan W. Hardy, Russell Carrington, Jonathan W. Kotula, Daniel A. Portnoy

**Affiliations:** 1 Department of Molecular and Cell Biology, University of California Berkeley 1438, Berkeley, California, USA; 2 Department of Plant and Microbial Biology, University of California Berkeley 1438, Berkeley, California, USA; 3 Department of Microbiology, Genetics, & Immunology, Michigan State University 3078, East Lansing, Michigan, USA; 4 Laguna Biotherapeutics, San Francisco, California, USA; Massachusetts Institute of Technology, Cambridge, Massachusetts, USA

**Keywords:** vaccines, *Listeria monocytogenes*, flavins, immunotherapeutics, MAIT cells

## Abstract

*Listeria monocytogenes* is a facultative intracellular bacterial pathogen that is a potent inducer of cell-mediated immunity, which has led to the development of attenuated, *Listeria*-based cancer vaccines. *L. monocytogenes* strains, such as live-attenuated double-deleted *Listeria* (LADD), lacking two key virulence factors, Δ*actA* and Δ*inlB*, have been used safely in clinical trials and showed promising anti-tumor activity. Despite early clinical success, improving potency and safety by preventing extracellular bacterial growth is paramount for the development of further clinical applications. We describe a quadruple attenuated intracellular *Listeria* (QUAIL) strain that, in addition to Δ*actA*Δ*inlB,* lacks *ribC* and *ribF*, which encode enzymes required for generating the essential flavin cofactors flavin mononucleotide (FMN) and flavin adenine nucleotide (FAD). QUAIL imported FMN and FAD during intracellular growth but was unable to grow extracellularly in blood or on vascular catheters in mice, which reduced its lethality. Despite its lack of extracellular growth, QUAIL maintained its immunoprotective properties, which were comparable to LADD. Furthermore, we showed that QUAIL can be engineered to synthesize riboflavin, leading to expansion and activation of mucosal-associated invariant T cells. Together, our data support the use of QUAIL as a promising therapeutic platform with an improved safety profile that is amenable to further modifications to expand its immune-activating potential.

**IMPORTANCE:**

*Listeria*-based live-attenuated cancer vaccines represent a promising therapy in many different pre-clinical tumor models and in clinical trials. Enhancing its anti-cancer immunity and increasing its safety profile will advance the clinical applications of *Listeria* vaccines. By manipulating *Listeria monocytogenes* flavin metabolism, we engineered a quadruple attenuated intracellular *Listeria* (QUAIL) vaccine candidate strain that has limited toxicity associated with extracellular growth in major extracellular niches *in vivo,* including blood and implanted catheter ports. Furthermore, we showed that QUAIL can be effectively programmed to engage innate-like T cells known as mucosal-associated invariant T cells, which could be harnessed for future cancer immunotherapies. The results presented here lay the foundation for further analysis of QUAIL as a safer, yet immunopotent *L. monocytogenes* vaccine or therapeutic vector.

## INTRODUCTION

*Listeria monocytogenes* is a gram-positive facultative intracellular bacterial pathogen that infects numerous cell types, escapes from phagosomes, and grows in the host cell cytosol. Cytosolic growth of *L. monocytogenes* leads to activation of an innate immune response that leads to induction of robust CD8^+^ T-cell-dependent adaptive immunity (1, 2). The immunopotency of *L. monocytogenes* makes it an ideal vaccine platform, and to date, multiple attenuated *L. monocytogenes* strains have been tested in clinical trials (3).

One of the most prominent *Listeria*-based vaccine strains is Δ*actA*Δ*inlB,* known as live-attenuated double-deleted *L. monocytogenes* (LADD) (4, 5). LADD is defective in cell-to-cell spread (Δ*actA*) and fails to enter some nonphagocytic cells, such as hepatocytes (Δ*inlB*). LADD is over 3-log attenuated in mice largely due to the lack of ActA, while the *inlB* deletion reduces hepatocyte toxicity. LADD expressing tumor antigens showed remarkable efficacy in pre-clinical models and modest results in clinical trials against multiple cancers (3, 6). However, there have been reports of occasional toxicity associated with *Listeria*-based cancer vaccines, including patients developing systemic listeriosis due to *L. monocytogenes* extracellular growth in blood and on catheters, which presents a major limitation to clinical development (7, 8). To improve the safety profile of *Listeria*-based immunotherapy, other attenuation strategies of existing LADD strains have been tested, including killed but metabolically active (KBMA) LADD (9) and recombinase-induced intracellular death (*Lm*-RIID) LADD (10). KBMA and *Lm*-RIID have limited replication ability *in vivo;* however, their immunopotency was less than LADD.

**Fig 1:**
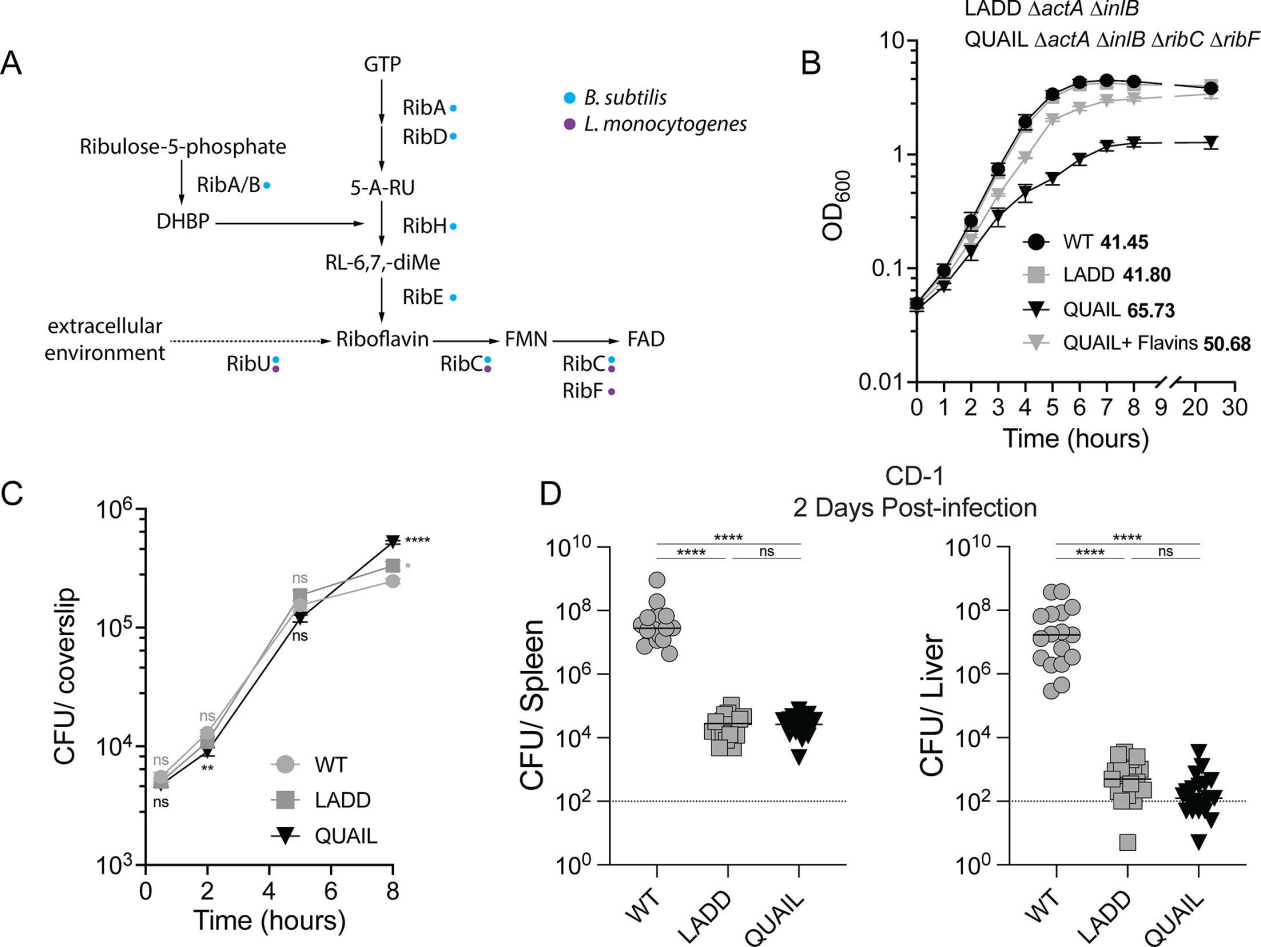
QUAIL and LADD display similar growth *in vitro* and *in vivo.* (**A**) Schematic of the riboflavin biosynthesis operon in *B. subtilis* (blue) and in *L. monocytogenes* (purple). *L. monocytogenes* is a riboflavin auxotroph and relies on extracellular sources of flavins that are imported by RibU (dotted line). *L. monocytogenes* △*ribC*△*ribF* is unable to produce FMN and FAD, and exogenous sources of FMN and FAD are required for survival. Intermediates in riboflavin biosynthesis: DHBP 3,4-dihydroxy-2-butanone 4-phosphate; 5-A-RU (5-Amino-6-(D-ribitylamino)uracil), RL-6,7-diMe 6,7-dimethyl-8-(d-ribityl)lumazine. Adapted from references 22, 23. (**B**) Broth growth curve of WT, LADD (△*actA*△*inlB*), and QUAIL (△*actA*△*inlB*△*ribC*△*ribF*) *L. monocytogenes* in BHI. QUAIL + flavins was grown in BHI supplemented with 2.5 μM FMN and 2.5 μM FAD. Optical density (OD_600_) was assessed over 24 h at 37°C with agitation. Mean ± SEM is shown, and data are pooled from two independent experiments. Bacterial doubling time is shown in bold. (**C**) Intracellular growth in bone-marrow-derived macrophages (BMMs) infected at an MOI = 0.25. Bacterial burdens (colony-forming units [CFUs]) were enumerated at the indicated times, and the results are mean± SEM from two independent experiments. One-way ANOVA, with multiple comparisons to WT, was performed. (**D**) CD-1 mice were infected intravenously with 10^5^ CFUs of the indicated strains. Spleens and livers were harvested 2 days post-infection, and bacterial burden was assessed by CFUs. Each data point represents an individual mouse, and lines represent medians. The dotted line is the limit of detection. Results are combined from five independent experiments with 17–20 mice per strain. One-way ANOVA, multiple comparisons. *P < 0.05, **P < 0.01, and ****P < 0.0001; ns, not significant.

Exploring auxotrophy as an attenuation strategy has been reported in different bacterial vaccines, including *Salmonella*, *Shigella,* and *Listeria* (11–14). *L. monocytogenes* is auxotrophic for a few amino acids and vitamins, including riboflavin (vitamin B2), which is essential for many metabolic redox reactions (15). *L. monocytogenes* acquires riboflavin and its essential flavin cofactor derivatives, flavin mononucleotide (FMN) and flavin adenine nucleotide (FAD) *via* the RibU transporter (16) (Fig. 1A). Additional enzymes involved in *L. monocytogenes* flavin metabolism are RibC, which mediates the phosphorylation of riboflavin to FMN and the adenylation of FMN to FAD, and RibF, which also catalyzes the adenylation of FMN to FAD (17–19) (Fig. 1A). In the absence of RibC and RibF, *L. monocytogenes* relies solely on exogenous sources of FMN and FAD, which are present at much higher concentrations inside mammalian cells compared to extracellular sources such as blood (20, 21). Consistent with this observation, *L. monocytogenes* Δ*ribC*Δ*ribF* mutants displayed no intracellular growth defect, whereas bacterial growth in extracellular niches, including blood, gallbladder, and gastrointestinal (GI) lumen, was limited (16). FMN and FAD auxotrophies (Δ*ribC*Δ*ribF*) lead to *L. monocytogenes* adopting an obligate intracellular lifestyle *in vivo*, which we hypothesized would be beneficial for developing safe *Listeria*-based vaccine strains with limited extracellular replication and dissemination.

*L. monocytogenes* riboflavin auxotrophy has in part been attributed to an immune evasion strategy, specifically the avoidance of recognition by mucosal-associated invariant T (MAIT) cells (16, 24, 25), a subset of evolutionarily conserved innate-like T cells present at various mucosal tissues and blood (26). MAIT cells detect intermediates of microbial riboflavin biosynthesis presented on MR1 molecules on the surface of host cells (27–29). Activation of MAIT cells results in cytotoxicity of infected target cells as well as immune cell activation and regulation of tissue homeostasis (30–33). A number of potential therapeutic applications of MAIT cells have been proposed for infectious diseases and antitumor therapy, as MAIT cell-dependent antitumor responses have been observed in some types of cancer (34, 35). Interestingly, *L. monocytogenes* engineered to synthesize riboflavin (Δ*actA*-*ribDEAHT*) led to a massive increase in MAIT cells and induced antitumor responses in a mouse cancer model (25), suggesting a potential approach to enhancing the immunopotency of *Listeria*-based cancer vaccines.

In this study, we examined whether attenuation mediated by the deletion of *ribC*/*ribF* improves the safety profile of LADD. We generated a Δ*ribC*Δ*ribF*Δ*actA*Δ*inlB* quadruple attenuated intracellular *Listeria* (QUAIL) strain and compared QUAIL and LADD in mouse infections and immunization models. Our data demonstrated that, unlike LADD, QUAIL displayed accelerated clearance and limited extracellular growth *in vitro* and *in vivo* and elicited protective immune responses comparable to LADD. We also showed that engineering QUAIL to express a heterologous riboflavin biosynthetic gene operon triggered the expansion of MAIT cells, which may further increase the potency of *L. monocytogenes* cancer therapy.

## RESULTS

### Deletion of *ribC* and *ribF* does not affect intracellular growth but reduces toxicity

We previously showed that the deletion of *ribC*/*ribF* does not affect intracellular growth of wild-type (WT) *L. monocytogenes* (16). To determine whether Δ*ribC*Δ*ribF* impacts LADD (Δ*actA*Δ*inlB*) growth and virulence *in vitro* and *in vivo*, we constructed LADD with *ribC*/*ribF* deletion resulting in a QUAIL strain. QUAIL exhibited a growth defect in rich liquid media compared to LADD, but the growth rates were enhanced by the addition of FMN and FAD to the media (Fig. 1B). The intracellular growth rate of QUAIL and LADD was similar to WT in murine BMMs, but as previously noted in Δ*ribC*Δ*ribF* strains, QUAIL grew to slightly higher numbers at 8 h post-infection (Fig. 1C) (16). During intravenous (IV) infection of CD-1 mice, QUAIL was severely attenuated and reached the same CFUs as LADD in spleens and livers at 48 h post-infection (Fig. 1D), indicating that QUAIL and LADD retained the same degree of attenuation.

Next, to characterize the toxicity of LADD and QUAIL, we switched to using BALB/c mice that are more permissive to *L. monocytogenes,* particularly in extracellular niches (36, 37). Mice were infected IV with different doses of WT, LADD, or QUAIL, and survival probability and corresponding weight change were determined over 14 days (Fig. S1A and B). Mice displayed increased sensitivity and change in body weight in response to WT and LADD doses greater than 8.2 × 10^3^ and 1.1 × 10^7^, respectively. Remarkably, no mortality was observed in mice infected with QUAIL at any dose below 3 × 10^8^ (Fig. S1A). The collected data were used to calculate the median lethal dose (LD_50_) of WT, LADD, and QUAIL in BALB/c and compared with LD_50_ in CD-1 mice (Table 1). LD_50_ of QUAIL exceeded the LD_50_ of LADD by twofold in CD-1 and at least by threefold in BALB/c mice (Table 1), indicating that QUAIL has reduced lethality compared to LADD.

**TABLE 1:** Analysis of median lethality (LD_50_)

	Median lethality (LD_50_)	
Strain	CD-1	BALB/c
WT	1.5 × 10^4^	1.76 × 10^4^
LADD	3.7 × 10^7^	8.2 × 10^7^
QUAIL	7.75 × 10^7^	>3 × 10^8^

### QUAIL has impaired extracellular growth

**Fig 2:**
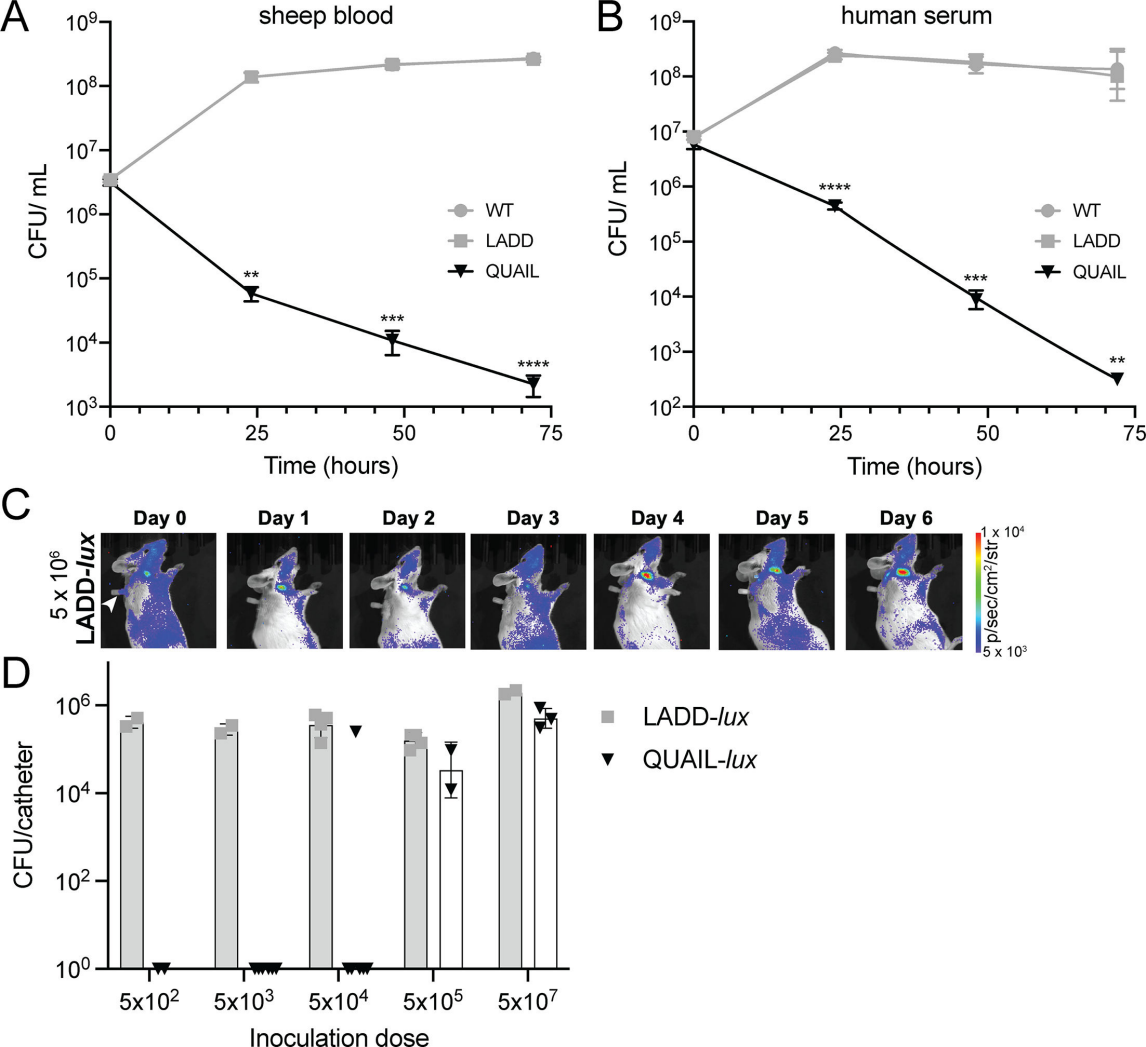
QUAIL does not grow in blood or on *in vivo* implanted catheters. Growth of WT, LADD, and QUAIL in 3 mL of sheep blood (**A**) or human serum (**B**) over the course of 72 h at 37°C with agitation. Bacterial counts were determined by plating for CFU. Results are the mean and SEM from three independent experiments. One-way ANOVA, multiple comparisons; **P < 0.01, ***P < 0.001, and ****P < 0.0001. (**C**) Bioluminescence imaging of CD-1 injected with 5 × 10^6^ CFUs of LADD-*lux* through the jugular vein catheter port (indicated with a white arrow). Mice were imaged using an *in vivo* bioluminescence imaging system (IVIS). A strong signal was detected on day 6 post-infection around the tip of the catheter below the anterior part of the neck. False-color scale bar indicated bioluminescence in photons/s/cm2/str. (**D**) Colonization of jugular vein catheters by LADD-*lux* and QUAIL-*lux*. CD-1 mice were injected with indicated doses of LADD-*lux* or QUAIL-*lux,* and catheters were collected on day 6 for bacterial enumeration by CFUs. Each data point represents an individual mouse. No CFUs were detected on catheters collected from mice injected with 5 × 10^2^ and 5 × 10^3^ of QUAIL-*lux* and in 4/5 mice injected with 5 × 10^4^ of QUAIL-*lux*.

During infection, a substantial proportion of *L. monocytogenes* reside in extracellular niches contributing to infectious burdens (38–41). Since our previous observations indicated that the deletion of *ribC*/*ribF* reduced the extracellular burden of WT *L. monocytogenes* during infection (16), we reasoned that the deletion of *ribC*/*ribF* would similarly prevent the extracellular growth of QUAIL. We first compared the growth of LADD and QUAIL in sheep blood and human serum. Unlike LADD that displayed increased growth during the first 24 h and maintained steady CFU levels between 24 and 72 h of growth, QUAIL displayed a continuous drop in CFUs in sheep blood and in human serum, suggesting that QUAIL is unable to survive in these conditions (Fig. 2A and B).

In addition to blood infections, data from clinical trials reported the growth of *L. monocytogenes* in extracellular niches, including implants and catheter ports (7). Considering that Δ*ribC*Δ*ribF* eliminates *L. monocytogenes* in extracellular niches such as blood, GI lumen, and gallbladder (16), we asked whether *ribC*/*ribF* deletion can also limit QUAIL growth on *in vivo* implanted catheters. Bioluminescent QUAIL-*lux* and LADD-*lux* were constructed by integration of the *lux* operon onto the *L. monocytogenes* chromosome (39). The bioluminescent strains were injected into the jugular vein catheter ports that were surgically implanted on the back of CD-1 mice. Initially, we aimed to directly examine bacterial burdens over time using an *in vivo* bioluminescence imaging system (IVIS). However, we noted that during extracellular growth, QUAIL-*lux* produced less signal intensity compared to LADD-*lux* (Fig. S2A and B). It is likely that QUAIL’s FMN/FAD auxotrophy negatively affected luciferase activity since it is dependent on FMN/FAD. The differences in bioluminescence signal between the two strains made it challenging to accurately compare the colonization of catheters using the IVIS. Instead, we used the IVIS to measure the colonization kinetics in individual animals to determine the optimal day to compare the CFUs of LADD and QUAIL strains in the catheters. Bioluminescence imaging was performed over the course of 6 days following jugular vein catheter injections with 5 × 10^6^ LADD (Fig. 2C). Bioluminescent signal was detected on days 0 and 1 following inoculation, after which the signal subsided on days 2 and 3, reappeared on day 4, and diminished on day 5, suggesting waves of *L. monocytogenes* replication and clearance as reported previously (39). A robust signal emanated from all catheters on day 6 post-infection, suggesting that day 6 was an appropriate time to examine bacterial growth on catheters (Fig. 2C). Mice were challenged with a range of QUAIL or LADD CFUs, and on day 6 post-inoculation, catheters were collected, vortexed in PBS, and bacterial burdens were examined by measuring CFUs from each individual catheter (Fig. 2D). Strikingly, unlike LADD that was present on catheters at all CFUs tested, QUAIL was undetectable on catheters after infection with 5 × 10^2^ or 5 × 10^3^ CFUs. Furthermore, four out of five mice had no bacteria on catheters when injected with 5 × 10^4^ QUAIL. QUAIL was present on catheters at infection doses above 5 × 10^5^, and the CFUs were comparable to LADD (Fig. 2D). Together, the data indicated that the *ribC*/*ribF* deletion reduced extracellular growth of QUAIL in multiple key extracellular niches, including blood and catheters.

### QUAIL is rapidly cleared in immunocompromised mice

**Fig 3:**
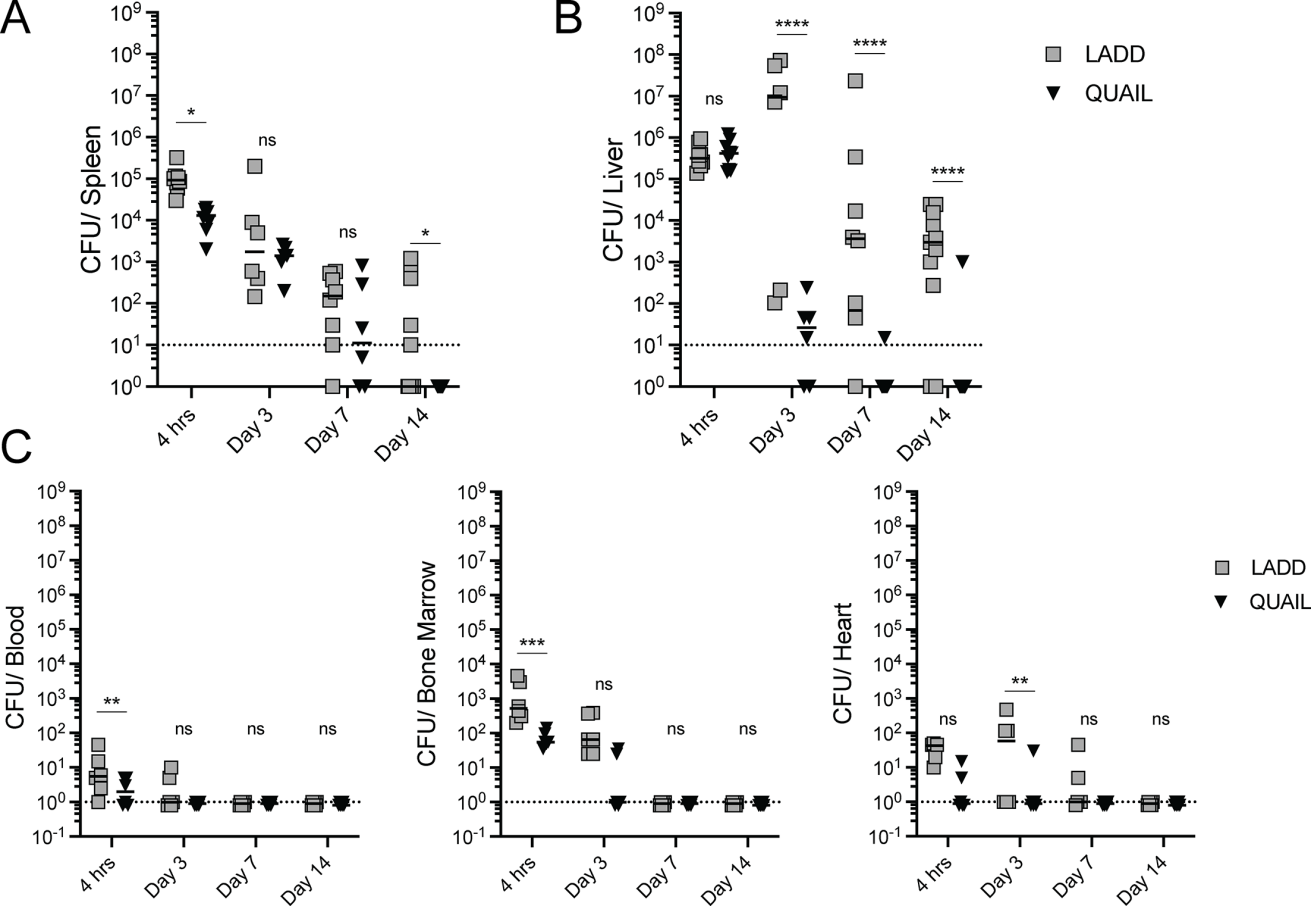
Enhanced clearance of QUAIL in Rag1^-/-^ mice. Rag1^-/-^ mice were infected IV with a dose of 10^6^ CFUs. Bacterial burdens of QUAIL and LADD were determined at the indicated time points in spleens (**A**), livers (**B**), and blood, bone marrow, and heart (**C**). Data were collected from at least two independent experiments with 6–12 mice per strain per indicated time point. The dotted line represents a limit of detection. Lines represent medians, and each data point represents an individual mouse. One-way ANOVA with multiple comparisons was used to compare CFUs at each time point between QUAIL and LADD infected groups; ns is not significant; *P < 0.05, **P < 0.01, ***P < 0.001, and ****P < 0.0001.

*L. monocytogenes* clearance in mouse IV models of listeriosis is mediated initially by host innate immunity during the first few days after infection and adaptive immunity at later times, which results in sterilizing immunity (42, 43). The loss of host immunity may lead to proliferation and subsequent delay of *L. monocytogenes* clearance *in vivo*, posing a risk for therapeutic use of live-attenuated cancer vaccines that are often administered to immunocompromised cancer patients. To determine whether the loss of anti-*Listeria* adaptive immunity compromises bacterial clearance, we measured the bacterial burdens of LADD and QUAIL in Rag1^-/-^ mice infected IV over the course of 14 days. Rag1^-/-^ mice lack B and T cells and have been used to study the role of immunity in mouse models of listeriosis (44). QUAIL was cleared more efficiently than LADD at 4 h and 14 days post-infection in the spleen (Fig. 3A). In the liver, LADD showed persistence, albeit a gradual decline in CFUs throughout the 14-day infection. Unlike LADD, QUAIL displayed rapid clearance in the liver with 4-log attenuation at 3 days post-infection and complete clearance after 7 days post-infection, suggesting that the persistence of LADD in the liver may be associated with extracellular bacteria (Fig. 3B). In addition to primary infection sites such as spleens and livers, *L. monocytogenes* can spread systemically *via* blood and lymph nodes and infect many organs including the GI tract, brain, and heart, leading to systemic listeriosis (45). Bacterial burdens of LADD and QUAIL were significantly lower in the systemic organs compared to CFUs detected in spleens and livers and were largely cleared 7 days post-infection (Fig. 3C). The highest detectable bacterial burden was observed soon after inoculation in blood and bone marrow. Accelerated clearance of QUAIL but not LADD occurred later during infection in blood, bone marrow, and the heart (Fig. 3C). Minor differences were detected in feces on day 3 post-infection, and most of QUAIL and LADD were undetectable in feces on day 3 post-infection (Fig. S3). No LADD or QUAIL were detected in the brain on day 7 and day 14 post-infection (Fig. S3). These results further demonstrated the superior safety features of QUAIL over LADD.

### QUAIL induces adaptive immunity and a potent CD8^+^ T-cell response

**Fig 4:**
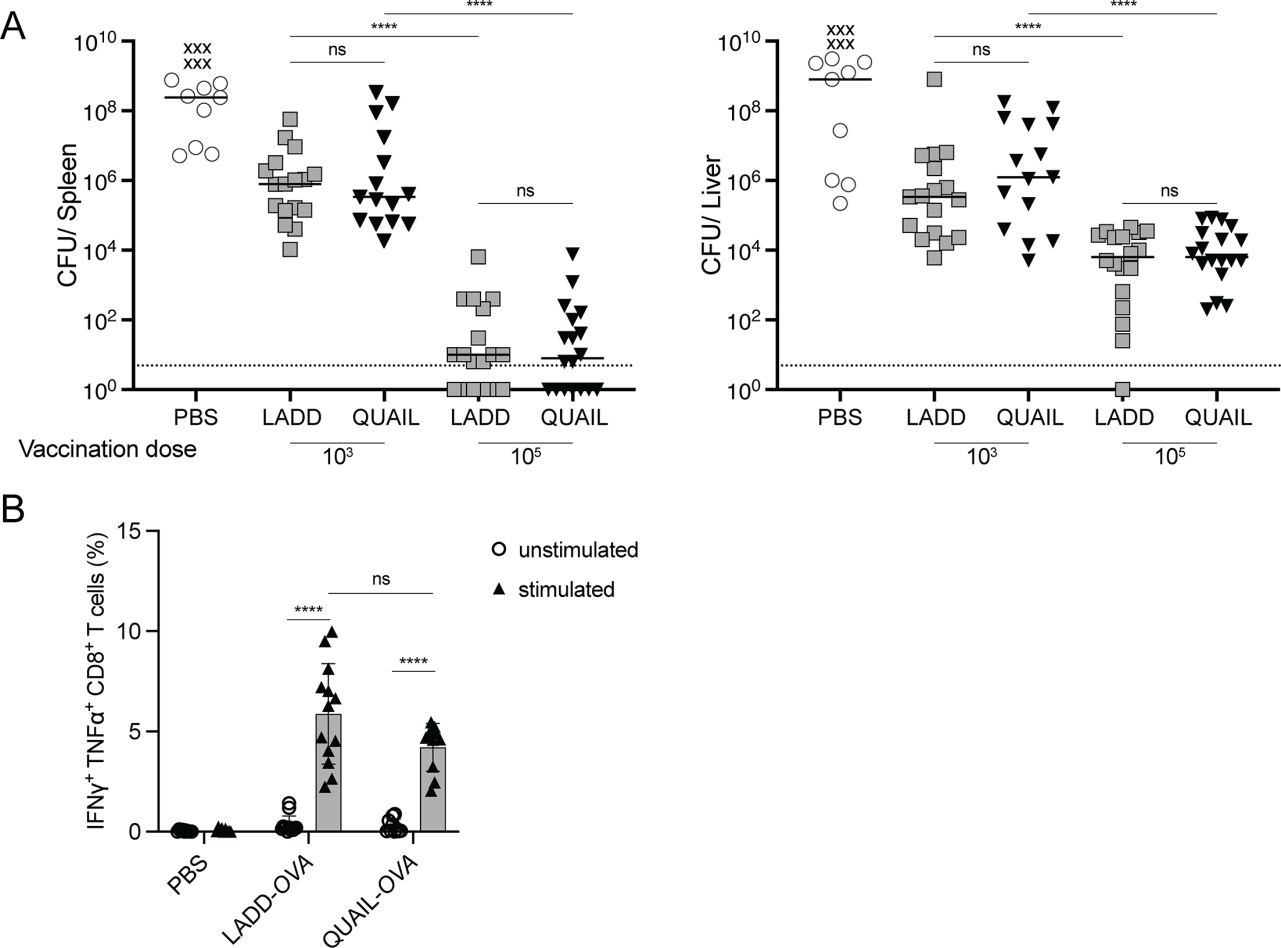
QUAIL and LADD stimulate comparable adaptive immunity. (**A**) Vaccination of C57BL/6J mice with 10^3^ or 10^5^ CFUs of LADD or QUAIL IV. 30 days post-vaccination, mice were challenged with 5 × 10^4^ of WT *L. monocytogenes* and spleens and livers were collected 3 days post-challenge. Results are combined from four independent experiments. Lines represent medians, and the dotted line indicates the limit of detection. Each data point represents an individual mouse. “x” represents mice that succumbed to infection. One-way ANOVA with multiple comparisons was used to calculate the significance. Ns is nonsignificant. (**B**) IV infection of C57BL/6J mice with 10^3^ CFUs of LADD or QUAIL expressing OVA. Splenocytes were collected 7 days post-infection, stimulated with OVA-specific peptide, and stained for intracellular cytokines. Data are combined from three independent experiments, means and SEM are shown. One-way ANOVA with multiple comparisons was used for statistical analysis. ns not significant, ****P < 0.0001.

To evaluate the relative capacity to induce protective immunity, we compared QUAIL and LADD using a C57BL/6J immunization model. Mice were immunized IV with either low (10^3^) or moderate (10^5^) doses of QUAIL or LADD. 30 days post-infection, mice were challenged with a lethal dose of WT *L. monocytogenes* as previously described (14, 46), and the CFUs in livers and spleens were determined 3 days post-challenge. At the moderate dose, both QUAIL and LADD induced better protection compared to lower dose vaccination in both spleens and livers (Fig. 4A). No significant differences were recorded between LADD and QUAIL immunization groups at each dose, and more potent protection was seen in the spleens compared to livers. These results suggest that immunization with QUAIL elicits an equally potent immune response as the well-characterized immunogenic LADD strain.

To further compare immunopotency of QUAIL and LADD, we examined the ability of the strains to induce CD8^+^ T cells. LADD- or QUAIL-expressing OVA was used to challenge C57BL/6J mice, and 7 days post-infection, splenocytes were collected and stimulated with OVA-specific peptides. The percentage of OVA-specific CD8^+^ T cells was determined by measuring CD8^+^ IFN-γ^+^ TNFα^+^ splenocytes by flow cytometry (Fig. 4B). QUAIL and LADD elicited comparable numbers of OVA-responsive CD8^+^ T cells, indicating that QUAIL and LADD are equally efficient at activating a cell-mediated immune response.

### Riboflavin-expressing QUAILs trigger expansion of MAIT cells

We recently showed that Δ*actA L. monocytogenes* expressing a *B. subtilis* riboflavin biosynthesis operon (*ribDEAHT*, Fig. 1A) (16, 18) leads to the activation of MAIT cells and can trigger antibacterial and tumor-restricting immunity (25). We hypothesized that the addition of the *ribDEAHT* operon into QUAIL and LADD can similarly lead to MAIT cell expansion, thereby enhancing immunopotency of the *L. monocytogenes* vaccine strains. Given that QUAIL is unable to grow extracellularly, we were curious to see whether *L. monocytogenes*-*ribDEAHT*-dependent activation of MAIT cells relies on bacterial ligands presented extracellularly, intracellularly, or both. QUAIL and LADD were engineered to express the *ribDEAHT* operon under the control of a constitutive promoter (P_hyper_). LADD-*ribDEAHT* was used as a positive control as we expected LADD to mimic the robust MAIT cell stimulatory properties of the previously reported Δ*actA*-*ribDEAHT* (25). First, we examined the growth *in vitro* and virulence *in vivo* of *ribDEAHT*-expressing strains. QUAIL-*ribDEAHT* and LADD-*ribDEAHT* showed no difference in growth compared to the corresponding parental strains in liquid media (Fig. S4A). The growth of LADD and LADD-*ribDEAHT* was indistinguishable in BMMs, and the CFUs of QUAIL and QUAIL-*ribDEAHT* were only marginally different at later stages of BMM infection (Fig. S4B and C). Together, these results indicated that the presence of the *ribDEAHT* operon did not have a significant impact on bacterial physiology or virulence *in vitro*.

**Fig 5:**
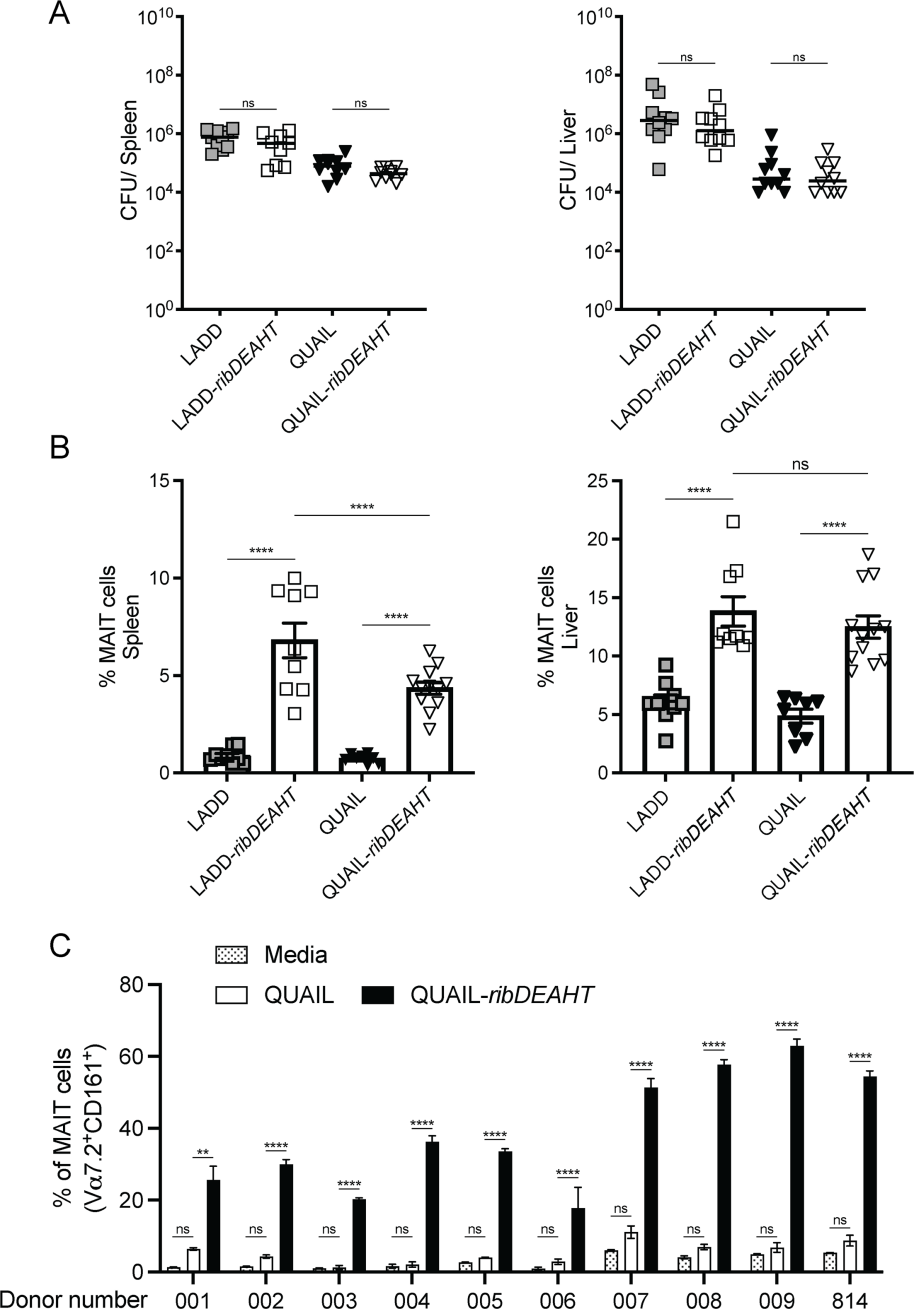
*ribDEAHT* expression in QUAIL and LADD triggers MAIT cell expansion. (**A**) CD-1 mice were infected with 10^7^ of the corresponding strains, and bacterial burdens were determined 2 days post-infection in spleens and livers. Results are combined from two biological repeats with 9–10 mice per strain. Each data point represents an individual mouse. One-way ANOVA, multiple comparisons. ns is not significant. (**B**) MAIT cell frequencies expressed as percentage of MR1^+^CD45^+^CD3^+^TCRβ^+^ from spleens and livers of C57BL/6J mice infected with 10^7^ *L. monocytogenes* strains for 4 days. Means and SEM are shown, data are pooled from four independent experiments with 9–12 mice per strain. One-way ANOVA was used to perform statistical analysis. (**C**) Frequencies of MAIT cells presented as percentage of Vα^+^CD161^+^ of αβ T cells from human peripheral blood mononuclear cells (PBMCs) infected with 2 × 10^9^/mL of *L. monocytogenes* strains for 1 h. PBMCs were supplemented with IL-2 at 1 h and at 4 days post-infection. 10 donors were used, and each donor sample was divided into replicate plates for infection with the indicated strains or media control. Experiments were performed twice in triplicate, and means and SEM are shown. Statistical analysis between strains for each donor was performed using one-way ANOVA. Ns is not significant, *P < 0.1, **P < 0.01, ****P < 0.0001. A detailed gating strategy is described in Fig. S5, and a detailed list of surface markers used for MAIT cell staining is provided in Table S1.

We recently reported that an IV infection with a high dose of 10^7^ CFUs of Δ*actA*-*ribDEAHT* led to MAIT cell expansion following a 4-day infection in mice (25). To verify that a similar sublethal 10^7^ dose of LADD- or QUAIL-*ribDEAHT* does not impact bacterial virulence, *ribDEAHT*-expressing strains were examined in an IV model of listeriosis in CD-1 mice (Fig. 5A). Both QUAIL- and LADD-*ribDEAHT* showed no virulence defect compared to their respective parental strains at 2 days post-infection. Of note, we did not see a decrease in bacterial burdens at 2 days post-infection with *ribDEAHT*-expressing strains compared to the parental strains, consistent with the observation that the MAIT cell-dependent reduction in bacterial CFUs was detected after 4 days post-infection (25). Infection with a 10^7^ dose resulted in LADD and LADD-*ribDEAHT* being 0.5–1-log more virulent in spleens and livers compared to QUAIL and QUAIL-*ribDEAHT* (Fig. 5A), despite both strains showing comparable virulence at a 10^5^ infection dose (Fig. 1D). The difference in virulence between the strains observed at 10^7^ but not at 10^5^ infection doses could be attributed to higher extracellular LADD burdens. Indeed, following 10^7^ infection doses, LADD and LADD-*ribDEAHT* burdens in the gallbladder, one of the key extracellular niches, were approximately 10^6^ CFUs 2 days post-infection, whereas QUAIL and QUAIL-*ribDEAHT* CFUs were below the level of detection (Fig. S4D).

Next, we examined whether *ribDEAHT*-expressing strains can trigger MAIT cell accumulation 4 days post-infection in C57BL/6J mice. MAIT cell frequency was determined using flow cytometry with the tissue samples stained with an MR1-restricted-5-OP-RU tetramer (Fig. S5A). The frequency of MAIT cells was less than 1% and 5% of all αβ T cells in spleens and livers following the infection with QUAIL and LADD, respectively. In contrast, infection with QUAIL- or LADD-*ribDEAHT* resulted in approximately a 5-7% and 14% increase in the frequency of MAIT cells of all αβ T cells in the spleens and livers, respectively (Fig. 5B). Infection with LADD-*ribDEAHT* led to a minor yet significant increase in MAIT cell accumulation in the spleens compared to QUAIL-*ribDEAHT*. Both QUAIL- and LADD-*ribDEAHT* were equally efficient at triggering MAIT cell expansion in the liver (Fig. 5B), suggesting that extracellular bacterial growth may not be required for robust MAIT cell activation in this organ.

Next, we examined the ability of *ribDEAHT*-expressing *L. monocytogenes* to expand MAIT cells from human PBMCs of healthy donors. Since QUAIL- and LADD-*ribDEAHT* exhibited a similar MAIT cell frequency during murine infection *in vivo* (Fig. 5B), we focused our analysis on QUAIL and QUAIL-*ribDEAHT* strains. PBMCs from 10 donors were infected with QUAIL or QUAIL-*ribDEAHT* for 1 h, and the media was supplemented with IL-2 to promote T-cell survival and differentiation. Cells were harvested 7 days post-infection for the analysis of MAIT cell frequency using flow cytometry (Fig. S5B). QUAIL-*ribDEAHT* triggered robust MAIT cell expansion potency compared to QUAIL-infected or uninfected cells across all donors (Fig. 5C). Collectively, these results suggested that QUAIL engineered to express riboflavin is effective at triggering MAIT cell accumulation both in mice and in human cells.

## DISCUSSION

In this study, we presented the construction and characterization of a live-attenuated *L. monocytogenes* strain referred to as QUAIL that is unable to grow extracellularly *in vivo*. We demonstrated that additional attenuation of a well-established Δ*actA*Δ*inlB* vaccine strain (LADD) was achieved by deleting *ribC* and *ribF,* rendering the bacteria dependent on host-derived sources of FMN and FAD. Both LADD and QUAIL induced protective immunity, but unlike LADD, QUAIL was unable to grow extracellularly and was rapidly cleared *in vivo*, offering a new avenue for developing safer vaccines and therapeutics.

The QUAIL vaccine platform presented here addresses the safety limitations associated with the use of LADD (3). QUAIL relies on exogenous FMN and FAD for growth, which limits the growth capacity of these strains in extracellular niches *in vivo* where FMN/FAD are limiting (47). QUAIL showed reduced toxicity (Fig. S1A; Table 1), enhanced clearance *in vivo* compared to LADD (Fig. 3), loss of bacterial viability in blood, and impaired colonization of catheters in mice (Fig. 2). The superior safety properties of QUAIL enhanced the safety potential of *L. monocytogenes* vaccines by several criteria. First, the QUAIL platform presents an opportunity to expand *L. monocytogenes* cancer vaccine clinical trials to involve patients with implanted medical devices, who were excluded from past and current trials, as they may be at risk of developing listeriosis linked to *L. monocytogenes* colonization and growth on ports, heart valves, prosthetics, and implants (7, 48). Second, the lack of extracellular growth of QUAIL *in vivo* may lead to re-evaluation of the current range of possible *L. monocytogenes* immunization doses that can be safely tolerated in patients (49). Lastly, the lack of growth in feces prevents QUAIL from spreading and potentially growing in the environment.

A number of previously evaluated attenuation strategies reduced bacterial growth but also reduced immune activation, demonstrating that there is a limit of attenuation that can be achieved without compromising vaccine efficacy (9, 10). Importantly, QUAIL and LADD induced similar CD8^+^ T-cell immune responses in the spleen and were equally effective at generating immune protection in immunization studies (Fig. 4). Despite attenuation through abrogated cell-to-cell spread and limited infection of hepatocytes (Δ*actA* and Δ*inlB,* respectively), LADD retains the ability to replicate extracellularly (Fig. 2). WT *L. monocytogenes* grows extracellularly *in vivo* in mesenteric lymph nodes, blood, gallbladder, and GI lumen (16, 37, 38, 50) and can also grow extracellularly following host cell lysis. Extracellular *L. monocytogenes* can activate the immune response locally and systemically (51). Since infection with LADD includes both extracellular and intracellular bacterial populations and QUAIL is predominantly intracellular, our results imply that the extracellular bacteria present during LADD infections do not contribute significantly to the generation of potent immune responses in an IV infection mouse model (Fig. 4). But extracellular bacterial growth contributes to increased toxicity, which was reflected by differences in LD_50_ (Table 1).

The observation that QUAIL was unable to survive in low FMN/FAD environments, including blood and catheters (Fig. 2), raises a question: what drives the loss of QUAIL viability? We speculate that low FMN/FAD levels lead to dysregulation of one or more bacterial proteins that require FMN/FAD, also known as flavoproteins (52). There are more than 34 flavoproteins in *L. monocytogenes* (19, 53) that play important roles in redox homeostasis, extracellular electron transfer (18, 54), metabolic regulation (55), and peptidoglycan biosynthesis (56, 57). Disruption of flavoprotein function may lead to bacterial death and possibly bacteriolysis, followed by an altered innate immune response in the extracellular environments (58). However, bacteriolysis may not be the main trigger for the loss of viability of QUAIL, since QUAIL exhibited no toxicity *in vivo* even at higher infection doses (Fig. S1A), suggesting that the clearance of the extracellular bacteria is not detrimental to the host. In the future, it will be important to define the molecular mechanisms that result in QUAIL loss of viability extracellularly and assess the degree of innate immune activation in response to QUAIL in both *in vivo* mouse models and in humans.

As well as improving safety, other studies have focused on increasing the potency of *L. monocytogenes* vaccines (3). Attempts to increase the versatility of *Listeria* vaccines include using *L. monocytogenes* as a vehicle for tumor-targeted delivery of radionucleotides or delivery of eukaryotic expression vectors encoding tumor antigens (59, 60). Our results showed that altering the metabolism of QUAIL to synthesize riboflavin led to expansion of MAIT cells *in vivo* (Fig. 5), as shown for Δ*actA*-*ribDEAHT* (25). QUAIL-*ribDEAHT* resulted in a robust increase in MAIT cell frequency comparable to LADD-*ribDEAHT* (Fig. 5), suggesting that MAIT cell expansion during *L. monocytogenes*-*ribDEAHT* infection may be independent of extracellular bacterial growth. However, we cannot exclude the possibility that during LADD-*ribDEAHT* infection, extracellular bacteria may also contribute to MAIT cell-specific ligand presentation, as different intracellular and extracellular pathogens have been shown to activate MAIT cells *via* distinct subsets of ligand-presenting cells (61). Whether the observed MAIT cell expansion induced by QUAIL-*ribDEAHT* infection translates into efficient anti-cancer immunity (62), as seen with Δ*actA*-*ribDEAHT* treatment (25), remains to be determined. *L. monocytogenes* is known to activate other innate-like T cells, such as γδ-T cells, *in vivo* and in tumor models (63). Examining the kinetics and functional characteristics of MAIT cells along with other innate-like T cells in different cancer models following QUAIL- and LADD-*ribDEAHT* treatment may advance our understanding of antitumor immune responses.

In conclusion, in this study, we showed that reprogramming *L. monocytogenes* flavin metabolism by deleting *ribC* and *ribF* limited the toxicity of *Listeria*-based vaccines by eliminating extracellular growth. In addition, by introducing the capacity to synthesize riboflavin, we expanded the diversity by which *Listeria*-based strains can activate MAIT cells. This observation presents an opportunity to use similar strategies to design *L. monocytogenes* vaccines capable of engaging with other unconventional T cells possessing antitumor potential, including γδ-T cells and invariant natural killer T cells.

## MATERIALS AND METHODS

### Bacterial strains construction and growth conditions

*L. monocytogenes* strains used in this study are listed in Table 2 and were derived from the WT parental strain 10403S. QUAIL strain was constructed by deleting the open reading frame of *ribC* (lmo1329) and *ribF* (lmo0728) in the Δ*actA*Δ*inlB* background (LADD). A detailed description of strain construction is in Supplemental methods.

**TABLE 2:** List of *L. monocytogenes* strains used in the study

Strain number	Background	Strain name	Reference
	*L. monocytogenes* 10403S	WT	(64)
DP-L7710	*L. monocytogenes* 10403S	LADD (∆*actA*, ∆*inlB*)	(4)
DP-L7712	*L. monocytogenes* 10403S	QUAIL (∆*actA*, ∆*inlB*, ∆*ribC*, ∆*ribF*)	This study
DP-L7669	*L. monocytogenes* 10403S	LADD-*lux* (∆*actA*, ∆*inlB, lux/kan*)	This study
DP-L7670	*L. monocytogenes* 10403S	QUAIL-*lux* (∆*actA*, ∆*inlB*, ∆*ribC*, ∆*ribF, lux/kan*)	This study
DP-L7666	*L. monocytogenes* 10403S	LADD-OVA (∆*actA*, ∆*inlB, pPL2-OVA*)	This study
DP-L7667	*L. monocytogenes* 10403S	QUAIL-OVA (∆*actA*, ∆*inlB*, ∆*ribC*, ∆*ribF, pPL2-OVA*)	This study
DP-L7711	*L. monocytogenes* 10403S	LADD-*ribDEAHT* (∆*actA*, ∆*inlB, pPL2x-Phyper-ribDEAHT*)	This study
DP-L7713	*L. monocytogenes* 10403S	QUAIL-*ribDEAHT* (∆*actA*, ∆*inlB*, ∆*ribC*, ∆*ribF, pPL2x-Phyper-ribDEAHT*)	This study

Bacterial strains were cultured in filter-sterilized brain heart infusion (BHI) medium (Becton Dickinson). Unless specified, QUAIL strains were grown in BHI supplemented with 2.5 µM riboflavin 5′-monophosphate (FMN, Millipore Sigma) and 2.5 µM flavin adenine dinucleotide (FAD, Millipore Sigma). Bacterial growth curves in BHI were performed using overnight bacterial cultures diluted in fresh media to an OD_600_ of 0.05. The strains were grown at 37°C with agitation (220 rpm), and growth was measured using a spectrophotometer. Bacterial doubling time was calculated between 2 and 5 h using pooled data from two biological repeats.

### Intracellular growth in BMMs

BMMs were collected from 8-week-old female C57BL/6J mice (The Jackson Laboratories) as described previously (65). BMMs were differentiated and cultured in BMM media containing DMEM (Thermo Fisher Scientific), supplemented with 20% FBS (Avantor-Seradigm), 10% macrophage colony-stimulating factor (M-CSF), 1% L-glutamine (Corning), 1% sodium pyruvate (Corning), and 14 mM 2-mercaptoethanol (Gibco Thermo Fisher Scientific). 3 × 10^6^ macrophages in BMM media were seeded on 60 mm non-tissue-culture treated dishes (MIDSCI), each containing 14 × 12 mm glass coverslips (Thermo Fischer Scientific), and grown overnight at 37°C. Bacteria were grown overnight without agitation at 30°C, and an MOI of 0.25 was used for infection. 50 μg/mL of gentamicin was added 1 h post-infection to remove extracellular bacteria. Bacteria CFUs were collected from three coverslips at each indicated time point.

### Bacterial growth in sheep blood and human serum

Growth in defibrinated sheep blood (HemoStat Laboratories) and sterile human serum (Sigma, H4522) was performed as previously described (16). Prior to experiments, human serum was heat-inactivated and buffered (pH 7 with 5 mM HEPES). Bacteria were grown at 37°C with agitation (220 rpm) until OD_600_ 0.5–1. The cultures were washed in PBS and resuspended in 3 mL of prewarmed blood to a concentration of 10^6^ CFU/mL, and cultures were incubated at 37°C, shaking for 72 h, and CFUs were determined by plating serial dilutions.

### Mouse infections

8- to 12-week-old female CD-1 mice (Charles River) were infected IV *via* the tail vein with 10^5^ CFUs of bacteria in 200 µL PBS. Animals were sacrificed 48 h post-infection, and spleens, livers (without gallbladder), and gallbladders were collected in 5 mL, 10 mL, and 0.2 mL of 0.1% IGEPAL (CA-630, Sigma) in water, respectively. Bacterial burdens were enumerated by plating serial dilutions.

8- to 12-week-old female Rag1^-/-^ mice in the C57BL/6 background (The Jackson Laboratories) were infected IV with 10^6^ CFUs of *L. monocytogenes*. Animals were sacrificed, and indicated organs were collected at 4 h, 3, 7, and 14 days post-infection. Spleens, livers (no gallbladders), and hearts were homogenized in 5 mL, 10 mL, and 2 mL of 0.1% IGEPAL in water, respectively. 500 μL of blood was collected following cardiac puncture after euthanasia, and 100 μL of 50 mM EDTA was added to prevent coagulation (66). Bone marrow was collected from the left femur and tibia, which were physically disrupted using a mortar and pestle and homogenized in 1 mL of 0.1% IGEPAL in water. Fecal pellets were weighed and homogenized in 1 mL of PBS. Homogenized tissues were plated using either serial dilution or 200 μL of undiluted for CFUs.

### Median lethality and body weight measurement

8- to 12-week-old female BALB/c or CD-1 mice were injected IV with gradual doses of the indicated strains, and survival and body weight were monitored over 14 days. A detailed description of the protocol is in Supplemental methods.

### Bioluminescence imaging using *in vivo* imaging system and bacterial collection from the catheters

LADD and QUAIL strains were made bioluminescent by transduction (67). A detailed description of the protocol used for imaging and CFU enumeration is presented in Supplemental methods.

### Vaccination of mice

8- to 12-week-old female C57BL/6J mice (The Jackson Laboratories) were immunized by IV injection with either 10^3^ or 10^5^ CFUs of *L. monocytogenes* in 200 µL PBS. 30 days post-immunization, mice were challenged IV with 5 × 10^4^ CFU of WT *L. monocytogenes*. Three days post-challenge, mice were sacrificed, and spleens and livers were harvested for homogenization in 0.1% IGEPAL (CA-630, Sigma). CFUs/organ were determined by plating serial dilutions or 200 µL of tissue homogenates.

### Analysis of antigen-specific T-cell responses

8- to 12-week-old female C57BL/6J mice (Jackson Laboratories) were injected IV with 10^3^ CFUs of *L. monocytogenes* in 200 µL of PBS. 7 days post-infection, single-cell splenocyte suspensions were stimulated with OVA 257-264 peptide epitope (SIINFEKL, InvivoGen vac-sin) in the presence of GolgiPlug protein transport inhibitor (BD Bioscience) for 4 h. The splenocytes were stained for viability, CD8 and CD4 markers, and fixed in 2% paraformaldehyde. Cells were permeabilized using Fixation/Permeabilization Solution Kit (BD Bioscience), and IFN-γ and TNF-α intracellular cytokine staining was performed using antibodies from eBioscience, listed in Table S1. Samples were analyzed by flow cytometry on an LSR Fortessa. Gating strategy included FSC-A and SSC-A to exclude cell debris and FSC-H and FSC-A to isolate single cells.

### Analysis of MAIT cell frequencies

8- to 12-week-old female C57BL/6 mice (The Jackson Laboratories) were infected IV with 10^7^ CFUs of *L. monocytogenes* in 200 μL of PBS. Spleens and livers were recovered 4 days post-infection. For *in vitro* analysis, PBMCs isolated from healthy human donors (STEMCELL Technology, Stanford) were resuspended in RPMI media supplemented with 10% FBS to 5 × 10^6^/mL. 2 × 10^9^ *L. monocytogenes* was used to infect 100 μL of cells per well in 96 U bottom for 1 h.

The detailed protocol is in Supplemental methods. The detailed gating strategy and surface markers used are described in Fig. S5 and Table S1.

### Statistical analysis

All statistical analyses were performed using GraphPad Prism version 10.0.1 for MacOS, GraphPad Software, La Jolla California, USA, https://www.graphpad.com/.

## SUPPLEMENTAL MATERIAL

Supplemental Material mbio.03652-25-s0001.docx Tables S1 and S2, Fig. S1 to S5, and supplemental methods.

ASM does not own the copyrights to Supplemental Material that may be linked to, or accessed through, an article. The authors have granted ASM a non-exclusive, world-wide license to publish the Supplemental Material files. Please contact the corresponding author directly for reuse.
