# Supplementary material for: Reprogramming Listeria monocytogenes flavin metabolism to improve its therapeutic safety profile and broaden innate T-cell activation
Source: mBio. 2025 Dec 31;17(2):e03652-25. doi: 10.1128/mbio.03652-25 (PMC12892937; doi:10.1128/mbio.03652-25)
Supplement: Supplemental Material — Tables S1 and S2, Fig. S1 to S5, and supplemental methods. [file mbio.03652-25-s0001.docx]

**Title: Reprogramming *Listeria monocytogenes* flavin metabolism to improve its therapeutic safety profile and broaden innate T-cell activation.**

**SUPPLEMENTAL MATERIAL**

**Supplemental Table S1**

**Supplemental Figures and Figure legends**

**Supplemental methods**

**SUPPLEMENTAL TABLE S1**

| **Tabe S1: Flow Cytometry cell staining markers** | | | |  |
| --- | --- | --- | --- | --- |
| Flow cytometry panel used in Figure 4B | | | |  |
| Viability | APC-Cy7 / BV510 | Invitrogen #L23105 | 1:1000 |  |
| CD4 | BV605 | Biolegend #100404 | 1:100 |  |
| CD8 | BUV737 | Biolegend #100704 | 1:100 |  |
| IFN-γ | FITC | Biolegend #505806 | 1:100 |  |
| TNF-α | PerCP-Cy5.5 / BB700 | Biolegend #506322 | 1:100 |  |
|  |  |  |  |  |
| Flow cytometry panel used in Figure 5B | | | |  |
| Viability | BV510 | Invitrogen #L23105 | 1:1000 |  |
| Thy1.2 | APC-Cy7 | Biolegend #202503 | 1:100 |  |
| CD45 | BUV395 | Biolegend #564225 | 1:100 |  |
| Tetramer | PE | NIH, Mouse 5-OP-RU tetramer PE labeled | 1:250 |  |
| CD3 | FITC | Biolegend #100306 | 1:100 |  |
|  |  |  |  |  |
| TCR-𝛾𝛿 | APC | ThermoFisher #17-5711-82 | 1:100 |  |
| TCR𝛽 | PerCP | ThermoFisher #45-5961-82 | 1:100 |  |
| Flow cytometry panel used in Figure 5C | | | |  |
| Viability | Near-IR | ThermoFisher L34993 | 1:1000 |  |
| CD4 | BV510 | Biolegend 317444 | 1:50 |  |
| CD8 | PERCP-Cy5.5 | Biolegend 344710 | 1:50 |  |
| CD3 | PE-Cy7 | Biolegend 300420 | 1:50 |  |
| Vɑ7.2 | PE | Biolegend 351706 | 1:50 |  |
| CD161 | APC | Biolegend 339912 | 1:50 |  |

**SUPPLEMENTAL FIGURES AND FIGURE LEGENDS**





**FIGURE S1**

Survival curves (A) and body weight change (B) of BALB/c mice infected IV with the indicated doses (CFUs) of WT, LADD and QUAIL. 5 mice per group were used per each strain. Mice were monitored daily for 14 days.

**FIGURE S2:** Bioluminescence of LADD-*lux* and QUAIL-*lux*

(A) Three independent colonies of *lux/kan*-expressing LADD-*lux* and QUAIL-*lux* were plated on BHI agar supplemented with FMN and FAD and imaged using IVIS Imagining system. ROI is region of interest. Mean signal intensity was calculated from three ROIs and shown below the image. LADD-*lux* is 2.3 times brighter than QUAIL-*lux* suggesting that *lux* reporter might be affected by disrupted flavin metabolism of QUAIL-*lux*.

(B) Mice were infected with 5 x 10^4^ of LADD or QUAIL and imaged using IVIS during the course of 6 days. The scale bar indicates the signal intensity in photons/sec/cm^2^/str. Images are representative of n=4 (LADD-*lux*) and n=5 (QUAIL-*lux*) infected mice.

**FIGURE S3:** LADD and QUAIL burdens in the brain and feces of Rag1^-/-^ mice

Bacterial burdens (CFUs) were determined at indicated time points following Rag1^-/-^ mice infection with LADD or QUAIL. The experiment was performed twice with 6-8 mice per strain per indicated time. Each point represents an individual mouse, the median is indicated by a bar, and the dotted line represents the limit of detection. One-way ANOVA with multiple comparisons was used to analyze the data. *P<0.1, ns is not significant.

Minor difference was recorded on day 3 post-infection in the feces, with no bacteria detected in the feces at 4 hours or on day 7 and 14 post-infection. No LADD or QUAIL were detected in the brain at day 7 and day 14.


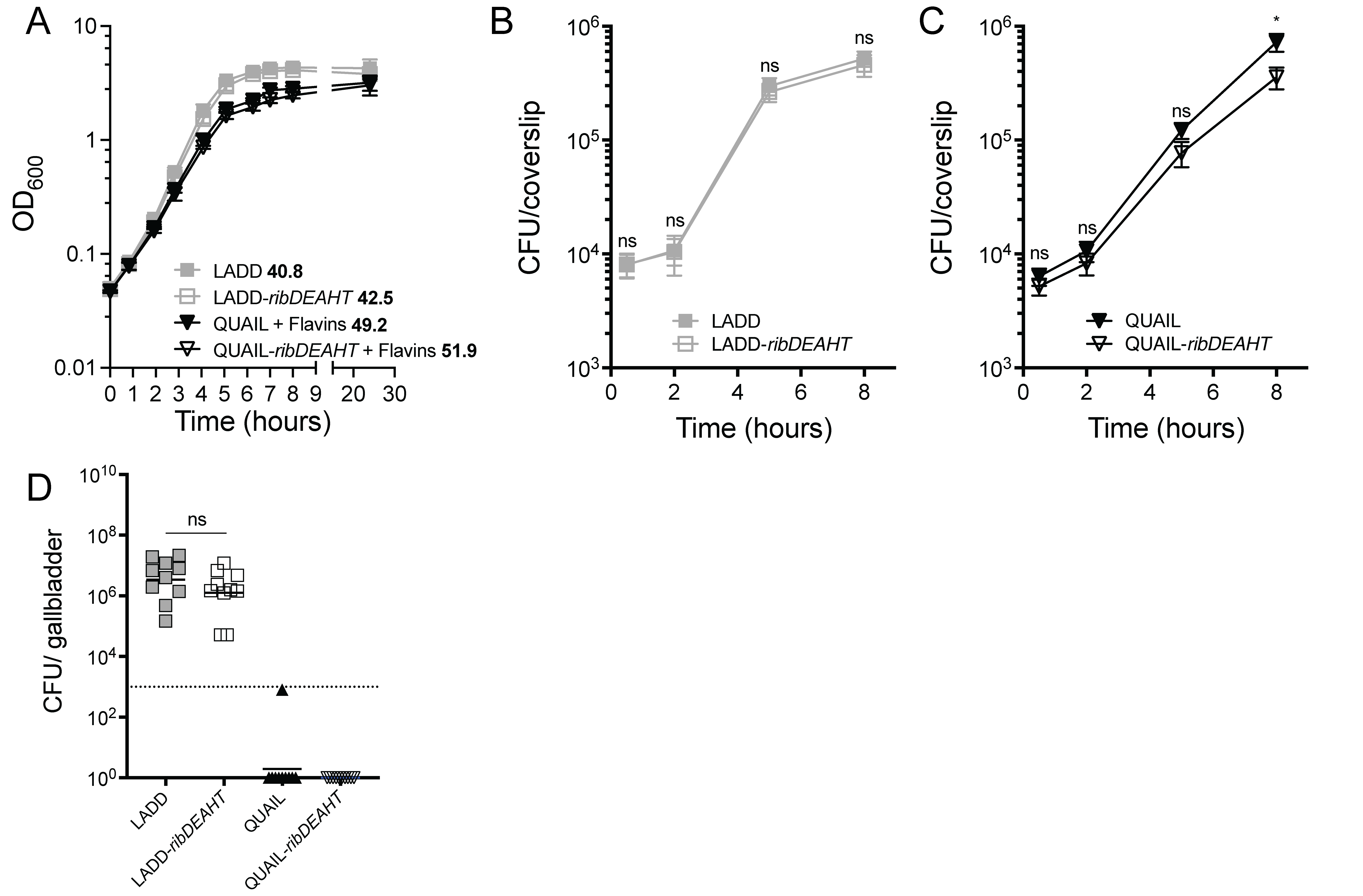


**FIGURE S4** Growth of *ribDEAHT*-expressing strains *in vitro* and in gallbladders.

(A) Growth of the indicated strains at 37^o^C in BHI broth with agitation. “+Flavins” indicate BHI supplemented with 2.5 M FMN and 2.5 M FAD. Optical density (OD_600_) was assessed using a spectrophotometer. The experiment was performed 3 times. Bacterial doubling time is shown in bold.

(B and C) Growth curves of the indicated strains in BMMs over the course of 8-hour infection. MOI of 0.25 was used for infection and gentamicin was added 1-hour post-infection to remove extracellular bacteria. Data represents 3 combined experiments and Mean SEM is shown. No significant difference was observed between LADD and LADD-*ribDEAHT* and minor but significant difference was observed at 8 hours between QUAIL and QUAIL-*ribDEAHT*.

(D) CD-1 mice were infected with 10^7^ of the indicated strains and bacterial burdens were determined 2 days post-infection in gallbladders. Results are combined from 2 biological repeats with 10 mice per strain. Each data point represents an individual mouse. Dotted line is the limit of detection.

Statistical analysis between strains was performed using One-way ANOVA, *P<0.05, ns not significant.


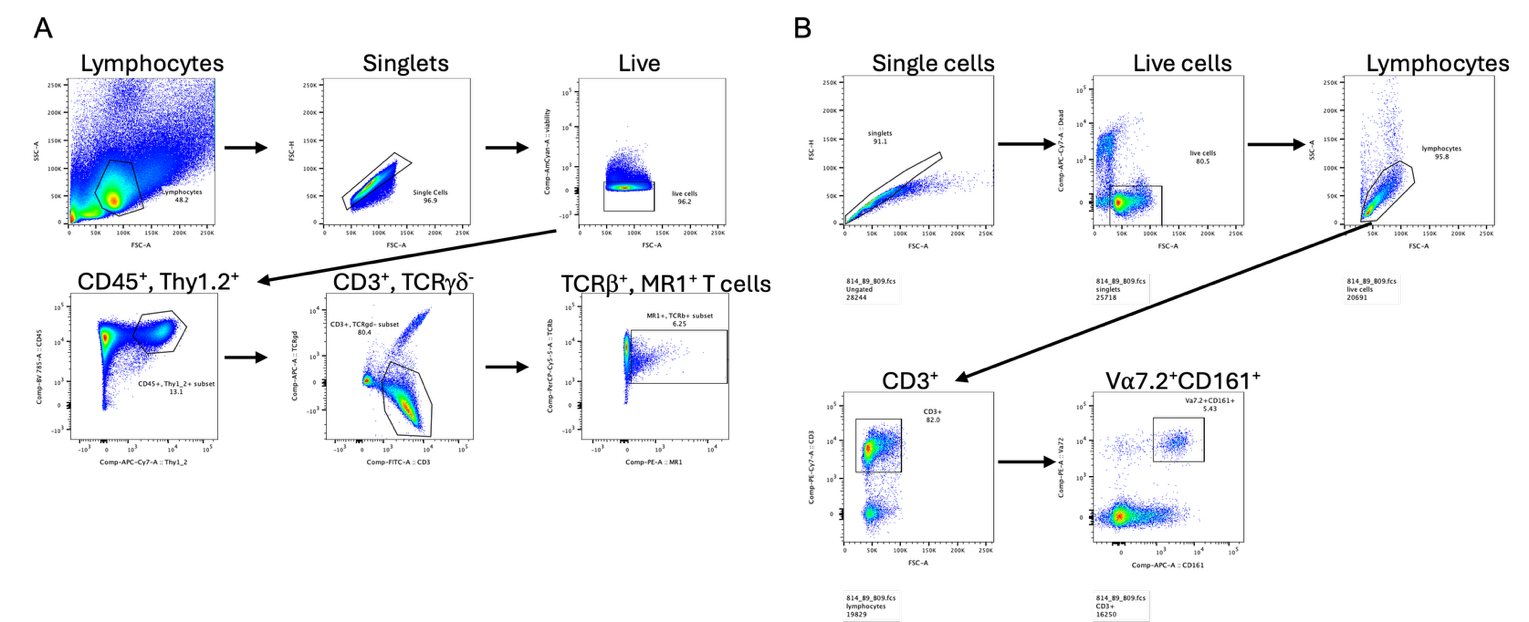


**FIGURE S5:** Representative gating strategies used for Flow cytometry analysis

(A) A representative FACS plot gating strategy used for data analysis presented in Fig 5B. Gating strategy depicts MAIT cell frequency (MR1^+^ cells from CD45^+^/CD3^+^/TCR^+^ population detected using MR1/5-OP-RU Tetramer, NIH) in the livers of C57BL/6J mice infected with 10^7^ LADD. Same strategy was applied for the analysis of splenocytes and liver cells collected from mice infected with LADD, LADD-*ribDEAHT,* QUAIL and QUAIL-*ribDEAHT*.

(B) A representative gating strategy used for the analysis of MAIT cells in Fig 5C. MAIT cell frequency was determined as percentage of V^+^CD161^+^ T cells in human PMBCs infected with QUAIL-*ribDEAHT*. The same strategy was used for the analysis of MAIT cell frequency in PBMCs infected with QUAIL or media control.

**SUPPLEMENTAL METHODS**

**Bacterial strains construction and growth conditions**

Gene deletion was performed using allelic exchange with pKSV7 vector as previously described (68). For *ribCribF* QUAIL construction, FMN and FAD-containing BHI media was used. OVA- and *ribDEAHT*- expressing strains were constructed by conjugating OVA-pPL2 (4, 5) or P_hyper_*ribDEAHT*-pPL2x (*ribDEAHT* operon was amplified from *B. subtilis* (16)) to *L. monocytogenes* using *E. coli* SM10 as a donor (69). *L. monocytogenes* transconjugants were selected on chloramphenicol (7.5 μg/mL, OVA-pPL2) or tetracycline (2 μg/mL, P_hyper_*ribDEAHT*-pPL2x).

**Analysis of MAIT cells frequencies**

Single cell splenocyte suspension was generated by addition of 2 mL of ACK (ammonium-chloride-potassium) lysis buffer (150 mM NH_4_Cl, 10 mM KHCO3, 0.1 mM Na_2_EDTA, pH 7.3). Livers were resuspended in 5 mL of Digestion mix RPMI 1640 with L-glutamine media supplemented with 25 mM 4-(2-hydroxyethyl)-1-piperazi-neethanesulfonic acid (HEPES), 20 mg/mL DNase I (Roche), and 125 U/mL collagenase D (Roche) for 1 hour at 37°C. Splenocyte and liver cell suspensions were passed through a 70 m nylon filter mesh FALCON, which was subsequently washed with 2 mL (spleen samples) and 20 mL (liver samples) of PBS. The resulting cell suspensions were centrifuged at 4^o^C for 5 min. Splenocyte cell pellet was resuspended in 1 mL of PBS. Liver cell pellets were resuspended in 2 mL ACK lysis buffer and incubated for 3 min at room temperature (RT). Following the addition of 4 mL PBS, cells were centrifuged for 5 min at 4^o^C and liver cell pellets were resuspended in 2 mL of PBS. The resulting single cell spleen and liver suspensions were added onto 96 well plate (100 L cell/well) and following centrifugation, the pellets were resuspended in 100 L of 0.5% FC blocking solution in PBS (Biolegend, #101320). The suspensions were incubated for 20 min at 4^o^C and centrifuged again. The cells were resuspended in antibody-tetramer mix and incubated for 30 min at RT. Following the incubation, cells were centrifuged for 5 min and washed twice with 200 L PBS. The cells were first fixed in 100 L TONBO fixation buffer (TNB-8222-L100) for 45 min at RT, then permeabilized with 50 L of TONBO perm buffer (TNB-1213-L150) and centrifuged again. The resulting pellets were resuspended in 150 l of TONBO perm buffer and stored at 4^o^C before imaging. Cell suspensions were washed and resuspended in 200 L of PBS and stained for viability using Live/Dead Fixable Blue (Molecular Probes) prior to Flow cytometry analysis.

For *in vitro* analysis, PBMCs were infected for 1 hour at 37^o^C and 5% CO_2_. The cells were washed with PBS and fresh media containing 100 μg/mL of gentamicin and 200 U/mL IL-2 (Fisher Catalog # 20002500UG) was added 1-hour post-infection and on day 4 post-infection. Cells were harvested on day 7 post-infection. MAIT cell frequencies were determined by flow cytometry using surface markers and antibodies listed in Table S1.

**Median lethality (LD_50_) and body weight measurements in BALB/c mice**

WT, LADD and QUAIL were grown until OD_600_1-1.5. Bacteria were pelleted, washed and resuspended in PBS containing 9% glycerol and stored at -80^o^C. On the day of infection, bacteria were thawed at room temperature and diluted to indicated concentrations in PBS prior to infection. CFUs/dose were determined by plating bacteria on BHI agar plates. For QUAIL, BHI was supplemented with flavins (2.5 µM riboflavin 5′-monophosphate (FMN, Millipore Sigma) and 2.5 µM flavin adenine dinucleotide (FAD, Millipore Sigma). 8–12-week-old female BALB/c mice (Charles River) were infected IV with the doses indicated in Fig S1A, B with 5 mice per group. Mice were monitored a minimum of two times daily (hourly the first day, transitioning to every 2 hours and then twice daily as animals stabilized) for signs of distress. Body weight was measured daily for the first 7 days, then transitioned to twice a week. Death was used as an endpoint unless mice were euthanized when they became moribund during monitoring. The criteria for moribund mice were: loss of mobility, hunched, signs of distress or pain and weight loss of more than 25%. Body weight change was calculated using the formula: (weight Day X – weight on Day 0 / weight on Day X). Infection, monitoring and data collection was performed by LumiGenics LLC.

**Median lethality (LD_50_) in CD-1 mice**

WT, LADD and QUAIL were grown overnight at 30^o^C without agitation in BHI. Overnight cultures were diluted in fresh BHI 1:10 and grown until mid-log OD_600_ 0.5-1. Indicated bacterial doses were prepared in PBS prior to infection. CFUs were determined by plating dilutions of bacterial cultures on BHI agar plates. 8-12-week-old female CD-1 mice (Charles River) were infected IV with the doses of WT, LADD and QUAIL listed in Table S2 where “n” indicates the number of mice used per group. Mice were monitored a minimum of two times daily for signs of distress. Mice were euthanized when they become moribund. The criteria for moribund mice were: loss of mobility, hunched, signs of distress or pain and weight loss of more than 15%.

**Table S2 *L. monocytogenes* doses and number of mice used to determine LD_50_**

| Bacterial strain | Dose (CFUs) and number of mice per group (n) | | | | | |
| --- | --- | --- | --- | --- | --- | --- |
| WT | 7.50 x 10^3^ (n=5) | 1.69 x 10^4^ (n=5) | 5.02 x 10^4^ (n=5) | 5.25 x 10^4^ (n=4) | 6.44 x 10^4^ (n=5) | 1.07 x 10^5^ (n=5) |
| LADD | 9.00 x 10^6^ (n=5) | 2.20 x 10^7^ (n=5) | 5.20 x 10^7^ (n=5) | 7.90 x 10^7^ (n=6) | 1.1 x 10^8^ (n=6) | n/a |
| QUAIL | 3.30 x 10^7^ (n=5) | 6.00 x 10^7^ (n=5) | 6.90 x 10^7^ (n=6) | 1.20 x 10^8^ (n=5) | 1.37 x 10^8^ (n=5) | n/a |

LD_50_ was calculated using Reed and Muench method as described previously (10)(70, 71) and presented in Table 1.

**Bioluminescence imaging using *In Vivo* Imaging System (IVIS) and bacterial collection from the catheters**

Phage U153 was collected from Xen32, a bioluminescent derivative of *L. monocytogenes* 10403S (the parental strain of LADD and QUAIL). The lysate was used to transduce the *lux/kan* insertion to QUAIL and LADD backgrounds and bacterial colonies were selected on BHI plates containing 50 g/mL kanamycin. 8–12-week-old CD1 mice with intravenous jugular vein catheters containing button ports with magnetic caps (Instech Inc.) were purchased from Charles River Laboratories. Following 1 day acclimation, the mice were injected through the catheters with heparin (500 units/50 L) using an Instech syringe. 48 hours post-heparin-injection, the mice were challenged with the indicated doses of LADD-*lux* or QUAIL-*lux* through the catheter using an Instech syringe. Imaging was performed over the course of 6 days post-infection using the IVIS instrument (Perkin Elmer Inc.). Mice were euthanized on day 6 and catheters were excised and imaged following dissection. The catheters were cut into 5 mm pieces, vortexed for 2 min in 10 mL of PBS and bacteria were enumerated by plating serial dilutions.

**REFERENCES**

68. Camilli A, Tilney LG, Portnoy DA. 1993. Dual roles of plcA in Listeria monocytogenes pathogenesis. Mol Microbiol 8:143-57.

69. Lauer P, Chow MY, Loessner MJ, Portnoy DA, Calendar R. 2002. Construction, characterization, and use of two *Listeria monocytogenes* site-specific phage integration vectors. J Bacteriol 184:4177-86.

70. Portnoy DA, Tweten RK, Kehoe M, Bielecki J. 1992. Capacity of listeriolysin O, streptolysin O, and perfringolysin O to mediate growth of *Bacillus subtilis* within mammalian cells. Infection and Immunity 60:2710.

71. Reed LJ, Muench H. 1938. A simple method of estimating fifty per cent endpoints. American Journal of Epidemiology 27:493-497.
